# DeepNGlyPred: A Deep Neural Network-Based Approach for Human N-Linked Glycosylation Site Prediction

**DOI:** 10.3390/molecules26237314

**Published:** 2021-12-02

**Authors:** Subash C. Pakhrin, Kiyoko F. Aoki-Kinoshita, Doina Caragea, Dukka B. KC

**Affiliations:** 1 School of Computing, Wichita State University, 1845 Fairmount St., Wichita, KS 67260, USA; scpakhrin@shockers.wichita.edu; 2 Glycan and Life Systems Integration Center (GaLSIC), Soka University, Tokyo 192-8577, Japan; kkiyoko@soka.ac.jp; 3 Department of Computer Science, Kansas State University, Manhattan, KS 66506, USA; dcaragea@ksu.edu; 4 Department of Computer Science, Michigan Technological University, Houghton, MI 49931, USA

**Keywords:** post-translation modification, sequon, deep neural network, N-linked glycosylation

## Abstract

Protein N-linked glycosylation is a post-translational modification that plays an important role in a myriad of biological processes. Computational prediction approaches serve as complementary methods for the characterization of glycosylation sites. Most of the existing predictors for N-linked glycosylation utilize the information that the glycosylation site occurs at the N-X-[S/T] sequon, where X is any amino acid except proline. Not all N-X-[S/T] sequons are glycosylated, thus the N-X-[S/T] sequon is a necessary but not sufficient determinant for protein glycosylation. In that regard, computational prediction of N-linked glycosylation sites confined to N-X-[S/T] sequons is an important problem. Here, we report DeepNGlyPred a deep learning-based approach that encodes the positive and negative sequences in the human proteome dataset (extracted from N-GlycositeAtlas) using sequence-based features (gapped-dipeptide), predicted structural features, and evolutionary information. DeepNGlyPred produces SN, SP, MCC, and ACC of 88.62%, 73.92%, 0.60, and 79.41%, respectively on N-GlyDE independent test set, which is better than the compared approaches. These results demonstrate that DeepNGlyPred is a robust computational technique to predict N-Linked glycosylation sites confined to N-X-[S/T] sequon. DeepNGlyPred will be a useful resource for the glycobiology community.

## 1. Introduction

Protein N-linked glycosylation is one of the most important post-translational modifications (PTM) that play essential roles in many vital biological processes like protein folding, protein stability, cell adhesion, molecular trafficking and clearance, receptor binding and activation, signal transduction, immune response, antigenicity, and apoptosis [1,2,3,4,5,6,7,8,9,10,11] in eukaryotes, archaea, and Gram-negative bacteria. Aberrant glycosylation is associated with the pathological progression of diseases such as SARS-CoV-2, Gaucher’s, Niemann-Pick type C, Sandhoff’s, congenital disorders of glycosylation (CDG), and Tay-Sachs [1,12,13,14]. N-linked glycosylation is a common feature shared by a significant fraction of transmembrane proteins, cell surface proteins, and proteins secreted in body fluids [1,15].

N-linked glycosylation involves the attachment of N-glycans (oligosaccharide) to the amine group (NH2) of asparagine (Asn(N)) and often occurs at the conserved motifs N-X-S or N-X-T sequons where X can be any amino acid except proline [6,16]. However, the presence of such sequon in the peptide does not sufficiently confirm that it is N-linked glycosylated because about one-third to half sequons are buried deep inside the proteins that are not accessible to glycosylation enzymes [17,18,19,20]. In addition, various factors like sequences surrounding a potential glycosylation site, distance to the next glycosylation, etc. site can impact whether the sequon is N-glycosylated or not. In that regard, the presence of sequon is necessary but not sufficient for N-linked glycosylation in both prokaryotes and eukaryotes [6,17,20,21].

Experimental techniques like mass spectrometry (MS) [22,23] and radioactive chemical labeling [24] are used to detect N-linked glycosylation. However, these techniques are time-consuming and labor-intensive. In this regard, several computational approaches to predict N-glycosylation sites have been developed. Some of the N-linked glycosylation site prediction approaches that use sequence-based features are: NetNGlyc [25] which uses an artificial neural network (ANN), EnsembleGly [26] uses an ensemble of support vector machines (SVM), GlycoPP uses a random forest (RF) [27], N-GlycoGo [28] uses XGBoost, Nglyc [29] uses RF, GlycoMine [30] also uses RF, SPRINT-Gly [31] uses Deep Neural Network (DNN) and others.

In addition to various sequence-based features, some approaches use predicted structural features like secondary structure (SS), accessible surface area (ASA), disordered regions, that are predicted by software like SABLE [32], PSIPRED [33], NetSurfP [34], SPIDER 3.0 [35], DISOPRED2 [36,37], SPOT-Disorder [38,39], etc. GlycoMine^struct^ [39] and SPRINT-Gly [31] are some of the approaches that use structure-based features in addition to sequence-based features. More recently, an SVM-based method to predict N-glycosylation site called N-GlyDE [40] that uses sequence features and predicted structural features was proposed.

It has to be noted here that with the exception of NetNGlyc and N-GlyDE all these approaches are evaluated on each N without being confined to N-X-[S/T] sequon although it was known for some time that the presence of this consensus sequon does not always lead to glycosylation [6]. Perhaps, this is the reason why the predictive performances of various approaches for predicting N-linked glycosylation site tend to be so high as the task here is to just predict each N in the N-X-[S/T] sequon as a glycosylation site. NetNGlyc and N-GlyDE are two seminal works in the field that exploit the fact that N-X-[S/T] sequon is necessary but not sufficient condition for glycosylation. More specifically, these approaches define the glycosylation site prediction problem as a classification problem to classify whether the given N-X-[S/T] sequon is likely to be glycosylated or not. Since N-GlyDE, a few predictors like N-GlycoGo [28], Nglyc [29] have confined their positive and negative datasets to N-X-[S/T] sequon. However, these predictors are trained on much smaller data sets and have not used other structural features.

Although N-GlyDE (a two-stage method) performs quite well, the predictive performance of N-GlyDE is not to the par as it can be used for high-throughput screening of computational glycosylation sites. Additionally, we have more N-Glycosylation sites due to advances in proteomics technologies, e.g., N-GlycositeAtlas database [41] contains more than 14,000 N-glycosylation sites from humans. Additionally, there exist no approaches for N-glycosylation (confined to the sequon) that have leveraged the modern Deep Learning algorithms. Recently, various DL-based approaches have been proposed in the field of bioinformatics [42,43,44,45].

Hence, we report DeepNGlyPred, a Deep Neural Network (DNN)-based approach to predict N-linked glycosylation site confined to N-X-[S/T] sequon. DeepNGlyPred uses various sequence-based features (e.g., Gapped-Dipeptide), predicted structural features (e.g., Secondary Structures, Accessible Surface Area, relative solvent accessibility (RSA), torsion angle (Φ, Ψ), disordered regions (calculated using NetSurfP-2.0 [46]), and PSSM (obtained using PSI-BLAST [47]).

DeepNGlyPred when trained using N-GlyDE training dataset and tested on N-GlyDE independent test set produces SN, SP, MCC, and ACC of 72.40%, 81.00%, 0.531, and 77.80%, respectively. In addition, it produces SN, SP, MCC, and ACC of 88.62%, 73.92%, 0.60, and 79.41%, respectively when trained on N-GlycositeAtlas training data and tested on N-GlyDE independent test set. These results demonstrate that DeepNGlyPred is a robust computational technique to predict N-linked glycosylation sites confined to N-X-[S/T] sequon.

## 2. Results and Discussion

In this section, we describe the results of DeepNGlyPred on the N-GlyDE dataset and N-GlycositeAtlas dataset. The datasets are described in details in Section 3.1 (Table 4).

### 2.1. Performance of DeepNGlyPred on N-GlyDE Dataset

Here, we describe the results of training DeepNGlyPred on the N-GlyDE training set and testing on N-GlyDE independent test dataset.

#### 2.1.1. Optimal Feature Set

**Figure 1:**
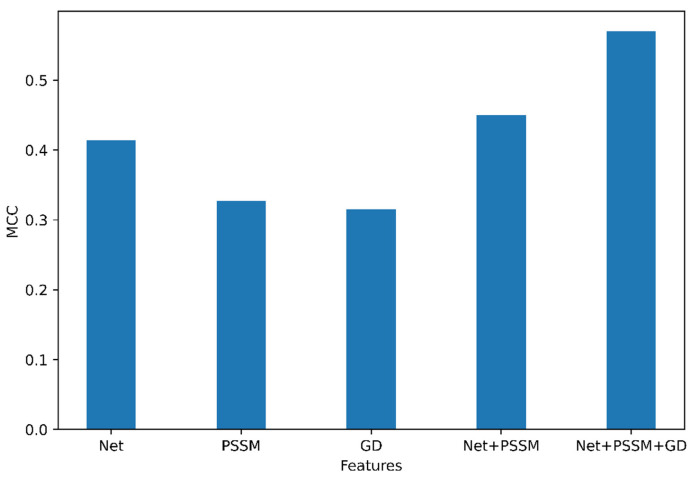
Individual and combined feature importance, Net represents features from NetSurfP-2.0, GD represents features from Gapped Dipeptide.

An optimal feature set can improve the performance of classifiers. In that regard, we performed an analysis to determine the optimal set of features for the classification of N-linked glycosylation sites. The results of this analysis are presented in Figure 1.

Among the group of features (NetSurfP-2.0, GD, and PSSM), the features obtained from NetSurfP-2.0 have the highest MCC (=0.41) in the independent dataset compared to PSSM and GD. Based on this, it can be seen that the feature combination of Accessible Surface Area (ASA), Relative Surface Area (RSA), Secondary Structure (SS), torsion angle (Φ, Ψ), disorder regions (obtained from NetSurfP-2.0) was the most important feature. Interestingly, Gapped Dipeptide was able to classify all the negative datasets as True Negative. The feature group was combined incrementally and as seen in Figure 1, the combination of features generated from NetSurfP-2.0, Gapped Dipeptide, PSI-BLAST (position-specific scoring matrix) provided the best MCC (0.57) from our model trained on the N-GlyDE dataset. Moreover, PSSM and Gapped Dipeptide (GD) performed similarly as shown in Supplementary Table S3.

Therefore, we can conclude that the feature combination of the Gapped Dipeptide, PSI-BLAST, and NetSurfP-2.0 is the best feature for prediction.

#### 2.1.2. Selection of Window Size

As NetSurfP-2.0 feature group was the most important among individual feature groups, we extracted the features generated from NetSurfP-2.0 for various window sizes such as 21, 23, 25, 27, and 29 (chosen based on the results from N-GlyDE) in an increment of 2 for the N-GlyDE dataset. The N-GlyDE training dataset was divided into 80% training, 10% validation, and 10% testing. The results of the performance of various window sizes for the 10% testing dataset are shown in Supplementary Figure S1. For the N-GlyDE dataset, the window size of 25 (as in N-GlyDE) (MCC: 0.336) resulted in the best performance which is elaborated in Supplementary Figure S1.

Based on these results, for the subsequent analysis for the N-GlyDE dataset, a peptide of window size 25 centered around the site of interest (N (Asn)) confined to sequon was created for both positive and negative N-linked glycosylation sites. Essentially, 12 amino acids upstream and downstream of central Asparagine (N) residue were extracted from the glycoprotein. When the site of interest was near the N-terminal or C-terminal of the protein, a virtual amino acid “-” was added to make the window sizes to be of the correct length.

#### 2.1.3. DNN Architecture

To determine the best performing Deep Neural Network architecture and parameters, a grid search was performed on a model with the N-GlyDE training dataset with three-fold cross-validation. This was done against 1, 2, 3, and 4 hidden layers, sigmoid and relu activation function, 30, 60, 90, 120, 150, and 240 neurons in each layer, RMSprop, and Adam optimizers, and 0.2, 0.3, 0.4, and 0.5 dropout rate, whereas default learning rate 0.001 was used. The optimized parameters using grid search are shown in Table 1.

#### 2.1.4. Cross-Validation Results

Ten-fold cross-validation was performed for the N-GlyDE dataset (3080 training examples). The mean MCC, mean Accuracy, mean Sensitivity, and mean Specificity for the ten-fold cross-validation were 0.4440 ± 0.0499, 0.7662 ± 0.0202, 0.9272 ± 0.0338, 0.4449 ± 0.1020, respectively. The detailed results of the 10-fold cross-validation are presented in Supplementary Table S1.

#### 2.1.5. Independent Test Results

When the trained model was applied on N-GlyDE independent test set, we obtained MCC, Accuracy, Sensitivity, and Specificity of 0.531, 77.80%, 72.40%, and 81.00% respectively. These results are slightly better than the performance of the N-GlyDE predictor. Additionally, while observing the confusion matrix of the DNN classifier the predictor was able to classify 227 as True Negative, 121 as True Positive. However, it falsely classified 53 as False Positive, and 46 as False Negative. All the individual and combined feature statistics are elaborated in Supplementary Table S3.

In addition, we also experimented with the dimension reduction technique based upon feature importance from XGBoost on the N-GlyDE dataset. The XGBoost algorithm was trained on the training set and then the threshold which is an important parameter was calculated. XGBoost resulted in the reduction of features from 3028 to 1188, and when those features were used to train the Deep Neural Network, we obtained efficiency score, MCC, Accuracy, Sensitivity, and Specificity, of 0.5059, 76.28%, 73.65%, 77.85%, respectively. The results of XGBoost are shown in Supplementary Table S2.

### 2.2. Performance of DeepNGlyPred on N-GlycositeAtlas Dataset

Here, we describe the results of training DeepNGlyPred on the N-GlycositeAtlas dataset.

#### 2.2.1. Optimal Feature Set

Three feature groups which are generated by NetSurfP-2.0, PSI-BLAST (PSSM), and Gapped Dipeptide performed the best in the N-GlyDE N-linked glycosylation dataset. Hence, the same feature groups were used to encode the positive and negative window sequence of the N-GlycositeAtlas dataset.

#### 2.2.2. Selection of Window Size

NetSurfP-2.0 feature group was the pivotal feature hence, we experimented with various window sizes such as 21, 23, 25, 27, 29, …, 41, 43, 45, 47, 49, and 51 in an increment of two for the N-GlycositeAtlas dataset. The N-GlycositeAtlas dataset was divided into 80% training, 10% validation, and 10% testing. We observed that the performing window size was 41 (highest MCC, 0.395) for the N-GlycositeAtlas test dataset. Hence, we utilize window size 41 for N-GlycositeAtlas dataset for subsequent experiments. We present the results of the performance of various window sizes in Supplementary Figure S2 and Supplementary Table S7.

For the N-GlycositeAtlas dataset, a peptide of window size 41 centered around the site of interest (N (Asn)) was created for both positive and negative N-linked glycosylation sites. Essentially, 20 amino acids upstream and downstream of the central Asparagine (N) residue constrained to sequon were extracted. When the site of interest was near the N-terminal or C-terminal of the protein, a virtual amino acid “-” was added to make the window sizes of the correct length.

#### 2.2.3. DNN Architecture

To obtain the best performing architecture and parameters, as in the case of the N-GlyDE dataset, we performed a grid search over Deep Neural Network Architecture. This was done against 3, 4, 5, and 6 hidden layers, sigmoid and relu activation function, 30, 60, 90, 120, 150, 240, 256, 512, and 1024 neurons in each layer, RMSprop, and Adam optimizers, and 0.2, 0.3, 0.4, and 0.5 dropout rate, whereas default learning rate 0.001 was used. The 3-fold cross-validation grid search produced an input layer with 1024 neurons, three hidden layers each with 1024 neurons, output layer consisting of two neurons with softmax activation function, dropout of 0.2, sigmoid activation function for input and hidden layers, and Adam optimizer with a learning rate of 0.001 as the best performing parameter for N-GlycositeAtlas dataset. The optimized parameters of DNN for the N-GlycositeAtlas dataset are elaborated in Table 2.

#### 2.2.4. Cross-Validation Results

Ten-fold cross-validation was performed on the N-GlycositeAtlas training dataset. We obtained MCC, SN, SP, and ACC of 0.5197 ± 0.0305, 0.6819 ± 0.0858, 0.8231 ± 0.00963, and 0.7532 ± 0.0127 for the 10-fold cross-validation. The results of 10-fold cross-validation are shown in Supplementary Table S1. The independent N-GlyDE test set result (elaborated in Section 2.2.5) produced by DeepNGlyPred when trained with N-GlycositeAtlas and 10-fold cross-validation results are similar which demonstrates that this model can be used for N-linked glycosylation prediction purposes.

#### 2.2.5. Independent Test Results

When the DeepNGlyPred trained on the N-GlycositeAtlas dataset was tested on N-GlyDE independent test set, we achieved MCC, SN, SP, ACC of 0.605, 88.62%, 73.92%, and 79.41% respectively. The DeepNGlyPred was able to classify 207 samples as True Negative, 148 samples as True Positive, 73 as False Positive, and 19 as False Negative.

### 2.3. Comparison of DeepNGlyPred with Other Machine Learning Models

**Figure 2:**
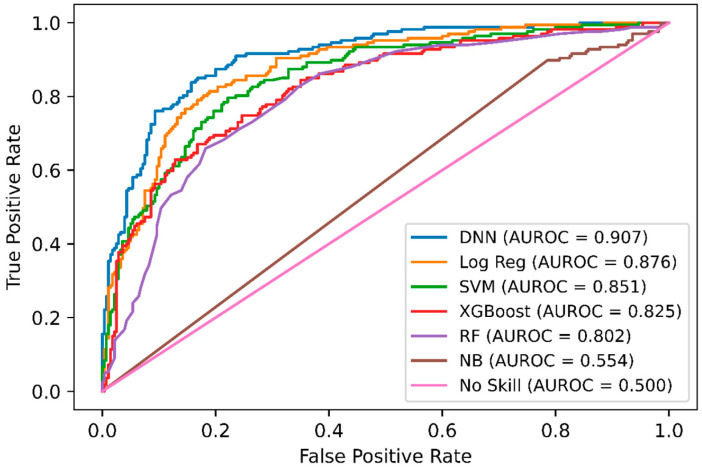
ROC curves of DNN, Logistic Regression, SVM, XGBoost, RF, and Gaussian Naïve Bayes on the independent dataset. For each model, the area under the ROC curve is reported.

**Figure 3:**
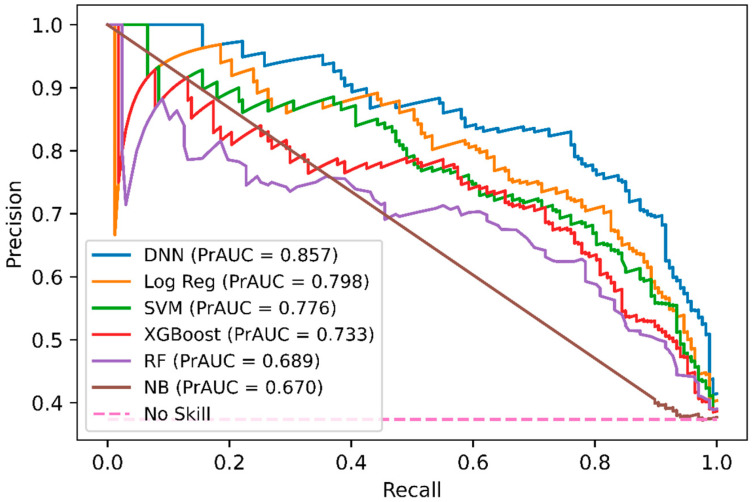
Precision-Recall curves of DNN, RF, SVM, Gaussian Naïve Bayes, Logistic Regression, and XGBoost on the independent dataset. For each model, the area under the Precision-Recall curve is reported.

In addition, we performed a comparison of DNN-based DeepNGlyPred with other optimized machine learning models like SVM, RF, Gaussian Naïve Bayes, logistic regression, and XGBoost. Figure 2 shows the receiver operating characteristic (ROC) curves for these models and our DNN-based model. All these models were trained on the N-GlycositeAtlas dataset and tested on N-GlyDE N-linked independent glycosylation datasets. Other performance metrics for these models are shown in Supplementary Table S5. Since the DNN model has the highest area under the curve (0.907) hence, DNN performs better than the other ML-based approaches. Precision and recall are used to assess the performance of a classifier on the positive class. So, we plotted the precision-recall curve of various machine learning models that are used to classify the N-GlyDE independent dataset. Figure 3 illustrates DNN has the highest precision-recall area under the curve (0.857), so, DNN can correctly classify the positive N-linked glycosylated sites better than other machine learning models.

### 2.4. Comparison of DeepNGlyPred with Other Deep Learning Models

**Figure 4:**
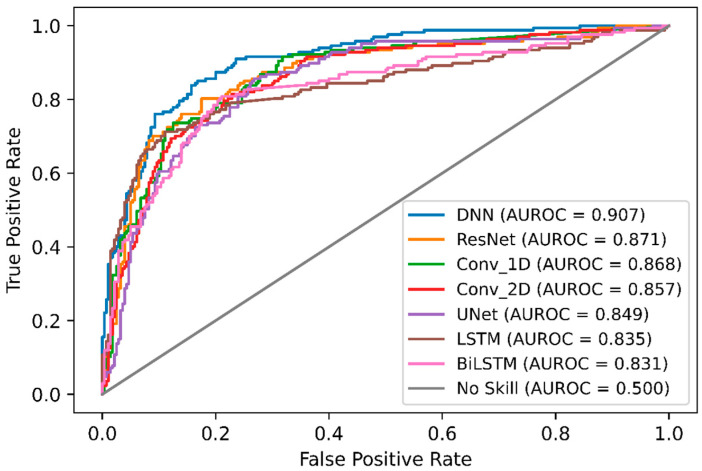
ROC curves of DNN, ResNet, Conv_1D, Conv_2D, UNet, LSTM, and BiLSTM on the independent dataset. For each model, the area under the ROC curve is reported.

**Figure 5:**
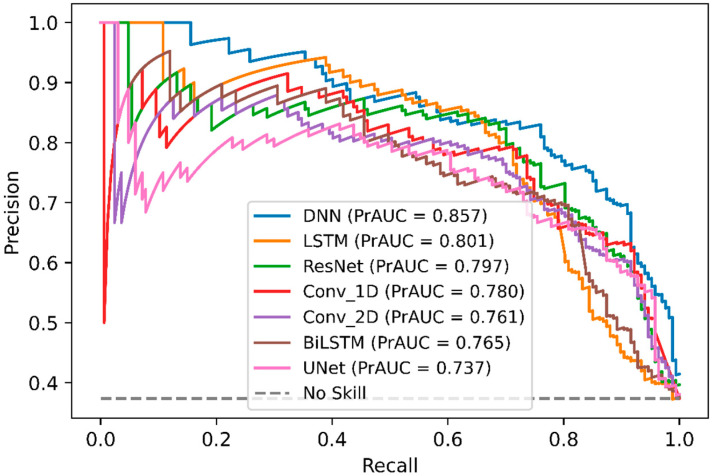
Precision-Recall curves of DNN, LSTM, ResNet, Conv_1D, Conv_2D, BiLSTM, and UNet on the independent dataset. For each model, the area under the Precision-Recall curve is reported.

In order, to compare DeepNGlyPred with other emerging deep learning models, we also implemented various other DL-architectures like Conv_1D, Conv_2D, LSTM, BiLSTM, ResNet, and UNet. The performance of these various DL-architectures is shown in Supplementary Table S6, and the ROC curve is shown in Figure 4. It can be observed from Figure 4 that DeepNGlyPred (based on MLP) has the highest area under the ROC. Figure 5 elucidates that DeepNGlyPred has the highest precision-recall area under the curve compared to other deep learning models, so DeepNGlyPred is a robust model for the prediction of N-linked Glycosylation PTM.

### 2.5. Comparison with Other Widely Available N-Linked Glycosylation Predictors

**Figure 6:**
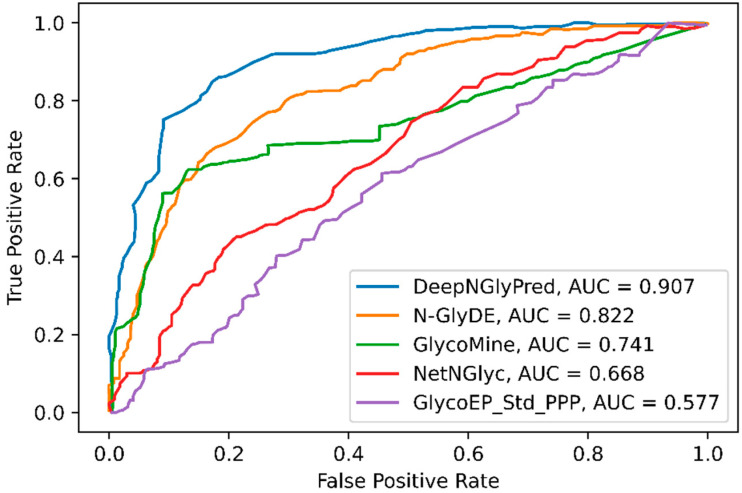
ROC curves of N-GlyDE, GlycoMine, NetNGlyc, and GlycoEP_Std_PPP on the independent dataset. For each predictor, the area under the ROC curve is calculated.

We further compared the performance of DeepNGlyPred with the publicly available predictors N-GlyDE, GlycoMine, NetNGlyc, and GlycoEP_Std_PPP and the results are shown in Table 3. Here, we trained DeepNGlyPred on the N-GlyDE and N-GlycositeAtlas dataset using the best set of features (obtained from NetSurfP-2.0, PSI-BLAST (PSSM), Gapped Dipeptide) and then tested on the N-GlyDE independent test set. The results for GlycoMine, N-GlyDE, NetNGlyc, and GlycoEP_Std_PPP were adapted from N-GlyDE [40]. It can be observed from Table 3 that DeepNGlyPred trained on N-GlyDE produced an MCC of 0.531, DeepNGlyPred trained on N-GlycositeAtlas produced an MCC of 0.605 compared to MCC of 0.499 for N-GlyDE. Furthermore, the independent test dataset was fed to SPRINT-Gly *N*- and *O*-linked glycosylation prediction webserver and the results are shown in Supplementary Table S8. As expected, the server classified all the positive and negative sequons as positive N-linked glycosylated sites. This result is evident because of differences in N-linked glycosylation site definition of SPRINT-Gly where sequon is defined as positive and Asparagine (N) other than positive site not confined to sequon are defined as negative. Additionally, we compared the various predictors using ROC-curve. Figure 6 illustrates, DeepNGlyPred has highest area under the curve (0.907) compared to other competitive predictors. From these results, it can be observed that DeepNGlyPred performs better than N-GlyDE and other methods compared.

## 3. Materials and Methods

### 3.1. Datasets

In this work, we used two benchmark datasets to train DeepNGlyPred. These datasets are the N-GlyDE dataset and the N-GlycositeAtlas dataset. 

#### 3.1.1. N-GlyDE Dataset

This dataset is adapted from N-GlyDE [40]. This dataset consists of 2050 experimentally verified N-glycosylation sites (N-X-[S/T] sequons) from 832 glycoproteins extracted from human Proteins in UniProt (ver. 201608). This is the set of positive glycosylation sites. Additionally, 1030 sites that follow the same N-X-[S/T] patterns from the same protein sequences but are not experimentally verified as N-glycosylation sites are considered as negative sites. This makes the training dataset.

As the sequons from subcellular compartments such as the nucleus, cytoplasm/cytosol, mitochondrion do not undergo N-linked glycosylation as reported by Zielinska et al. [18], in N-GlyDE 33 non-glycoproteins are selected to create non-glycosites. Additionally, 53 glycoproteins are chosen to create the set of glycosites. These 53 glycoproteins and 33 non-glycoproteins (after some filtering) have 447 N-X-[S/T] sequons (167 glycosites and 280 non-glycosites) which make the independent dataset.

#### 3.1.2. N-GlycositeAtlas Dataset

The other benchmark dataset considered in this work is based on N-GlycositeAtlas. N-GlycositeAtlas [41] is a recently developed large-scale repository for N-linked glycosylation that contains 7204 glycoproteins. It must be noted here that all the N-glycosylation sites of N-GlyDE are included in the N-GlycositeAtlas database. We downloaded the sequences of these 7204 (19 were obsolete) glycoproteins from the UniProt. Subsequently, we extracted 12,534 annotated positive sites from N-GlycositeAtlas confined to N-X-[S/T] sequons from these glycoproteins.

For the creation of negative sites, based on the knowledge that proteins from the nucleus and mitochondria are known not to undergo N-linked glycosylation [18], we obtained 5265 non-glycoproteins (from nucleus and Mitochondrion) using DeepLoc-1.0 [48] database. The N-X-[S/T] sequons from these proteins were extracted and after redundancy removal, we obtained 17,110 N-X-[S/T] sequons. Since there is a propensity of machine learning and deep learning algorithms to exhibit bias towards the majority class, we used an under-sampling [49] strategy to balance the dataset by randomly selecting negative sets to match the number of positive sites. This resulted in selecting 12,534 glycosites and 12,534 non-glycosites. Furthermore, we removed peptides that have more than 30% sequence identity using CD-HIT [50], we received 9450 positive sites and 10,363 negative sites. The negative sites were under-sampled to 9450 sites (Table 4). It also must be noted that none of the N-GlyDE independent test data is present in the N-GlycositeAtlas training dataset.

Based on the general belief that neighboring residues can influence the glycosylation status of the site of interest (in this case Asparagine (N) residues), a window centered around N and symmetrically surrounded by flanking residues on both sides (upstream and downstream) is generally taken as input. The number of upstream and downstream residues considered in creating this window is important because too few residues may omit useful information for making predictions. At the same time, too many residues may introduce ineluctable redundancy and decrease the signal-to-noise ratio. The most appropriate window size for each type of PTMs remains elusive, and most researchers test different fragment sizes and choose the one that produces the best predictive performance.

#### 3.1.3. WebLogo Plot

**Figure 7:**
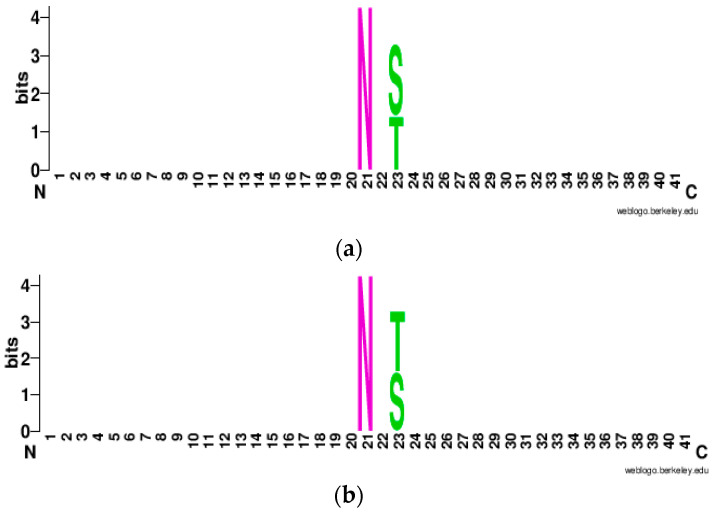
WebLogo plot: (**a**) Positive N-linked Glycosylation; (**b**) Negative N-linked Glycosylation.

To visualize the enrichment and depletion of amino acids in the corresponding position of the 41-window sequence, we create WebLogo [51] plots which are shown in Figure 7a,b. It can be observed that Asparagine (N) is conserved at the center whereas Serine (S) and Threonine (T) are conserved at the second position to the downstream of the central residue Asparagine (N). Furthermore, there were variable amino acids at other positions which had the lowest information content (bits), hence amino acids were not conserved at upstream (left) and downstream (right) of conserved Asparagine (N), Serine (S), and Threonine (T) site.

#### 3.1.4. t-SNE Plot

**Figure 8:**
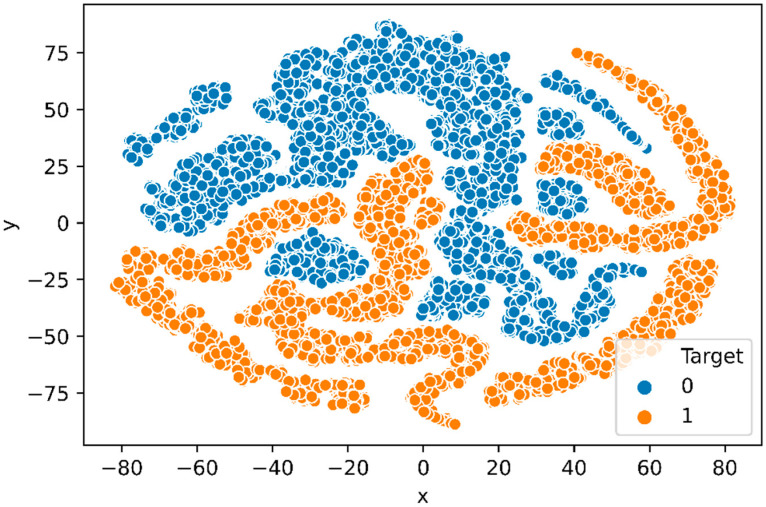
t-SNE plot: Feature vector generated from the final hidden layer of DNN trained with N-GlycositeAtlas dataset. The positive and negative samples are distinct in the t-SNE plot.

To investigate if the network has learned to encode the three combined feature groups in its representations, we project the feature vector produced by the final hidden layer of the trained deep neural network into two dimensions latent feature vector with popular dimensional reduction and visualization technique, t-distributed stochastic neighbor embedding (t-SNE) [52] method. Figure 8 represents the t-SNE plot, of the feature vectors generated from the final hidden layer of the deep neural network, which indicates that the deep neural network largely clusters positive and negative samples in ℝ^2^ space.

### 3.2. Features Used in DeepNGlyPred

Here we briefly describe the features used in DeepNGlyPred.

#### 3.2.1. Position-Specific Scoring Matrix (PSSM)

**Figure 9:**
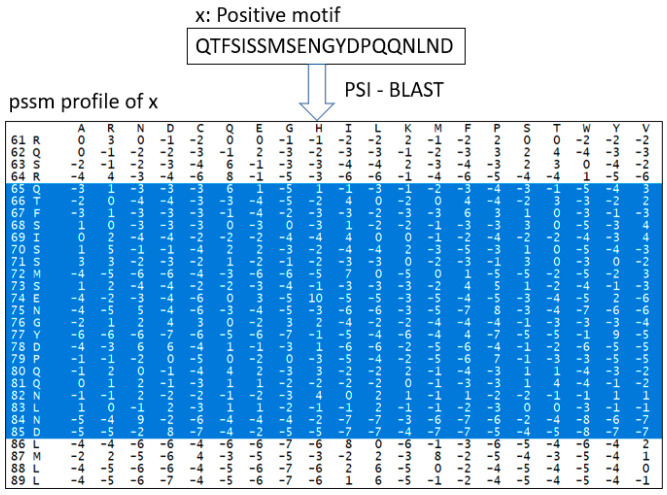
Feature vector construction of sequence using PSI-BLAST, all the selected features are flattened, min-max scaled, and fed into DNN.

PSSM is one of the features used in DeepNGlyPred. Based on the knowledge that N-linked glycosylation is more evolutionary conserved than other PTMs, we use PSSM features for N-linked glycosylation prediction. PSSM expresses the patterns inherent in a multiple sequence alignment on a set of homologous sequences. Furthermore, it renders a set of probability scores for each amino acid (or gap) at each position of the alignment. The PSSM matrix was generated by PSI-BLAST [47], wherein a full-length protein sequence was fed into PSI-BLAST for the generation of a PSSM file. The corresponding feature vector was extracted from the PSSM file based on the window size. For an amino acid, PSSM has 20 features so, for a window size of 41 (in the case of the N-GlycositeAtlas benchmark dataset) a feature vector of 820 (=41 × 20) is generated and a feature vector of 500 is generated for the N-GlyDE dataset. The virtual amino acid position is filled with the median of the column. Finally, the features are min-max scaled with the sklearn module and fed into the DNN model for training. For example, how the feature vector of sequence “QTFSISSMSENGYDPQQNLND” (length 21) is extracted is depicted in Figure 9.

#### 3.2.2. Predicted Structural-Features

Based on the observation that protein structural features provide additional information, we also consider the following predicted structural features in DeepNGlyPred.

##### Predicted Secondary Structure (SS)

We utilize NetSurfP-2.0 to predict three types of secondary structures, E (beta-strand), H (helix), and C (coil) and assign each amino acid one secondary structure based upon its propensity towards them. These three-class secondary structures are then one-hot encoded as E (beta-strand): [0, 1, 0], H (Helix): [0, 0, 1], C (Coil): [1, 0, 0], respectively. The overall protein sequence is fed to NetSurfP-2.0 and the corresponding features are extracted based on the window size. The feature vector length of the secondary structure feature for the N-GlycositeAtlas dataset is 123 (41 × 3) and for the N-GlyDE dataset is 75 (25 × 3).

##### Predicted Accessible Surface Area (ASA), Relative Solvent Accessibility (RSA)

Additionally, NetSurfP-2.0 also predicts RSA and ASA. The RSA value ranges from 0–1 where 0 means the amino acid is completely buried and 1 means it is completely accessible. RSA is a normalized version of ASA. We use both features in DeepNGlyPred and each amino acid gets one value of ASA and one value of RSA. Hence, the length of the resultant feature vector is 82 for N-GlycositeAtlas and 50 for the N-GlyDE dataset.

##### Predicted Disordered Region

Intrinsic disorder regions of the proteins do not have rigid three-dimensional structures and are usually found at loops and turns which are the target of PTMs. We predict disordered regions for each amino acid using NetSurfP-2.0. The predicted disordered region value ranges from 0 to 1 where 0 signifies ordered amino acid and 1 represents a completely disordered amino acid. The feature vector length based on the predicted disordered region is equal to the size of the window (41 for N-GlycositeAtlas and 25 for the N-GlyDE dataset).

##### Torsion Angles (Φ, Ψ)

The torsion angle between neighboring amino acids provides the local structure of a polypeptide. Φ and Ψ reveal the torsion angles between the molecules inside one single amino acid to the neighboring molecules. Torsion angles show structure near the locality of N-linked glycosylation sites. We predict torsion angles (=2) for each amino acid using NetSurfP-2.0. The feature vector length based on the torsion angle is 82 for N-GlycositeAtlas and 50 for the N-GlyDE dataset.

#### 3.2.3. Gapped Dipeptide (GD)

**Figure 10:**
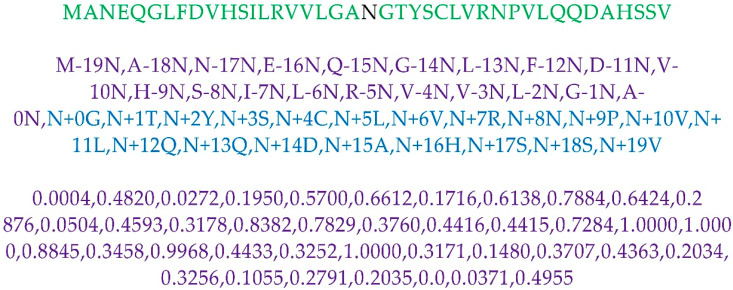
Schematic of Gapped Dipeptide Feature.

Gapped Dipeptide is one of the features used in N-GlyDE. For simplicity, we describe Gapped Dipeptide using the best window size for the N-GlycositeAtlas dataset. To compute Gapped Dipeptide, the integer distance from the N-terminal and C-terminal of the peptide to the Central residue Asparagine (N) is considered for every amino acid in the positive and negative 41-residue window. Furthermore, it can be expressed as AkN (0 ≤ k ≤19) for the N-terminal and NkA (0≤ k ≤19) for the C-terminal 41-residue window, where A is one of the twenty amino acids or virtual amino acid (“-”). Figure 10 shows the schematic of the Gapped Dipeptide feature. From the 18,900 41-residue window peptides (N-GlycositeAtlas training dataset), we obtained 822 gapped dipeptide features. The number of occurrences of each gapped dipeptide in glycosites and non-glycosites is counted. After that, each gapped dipeptide odds ratio is calculated by the percentage of its occurrences in glycosites divided by the percentage of its occurrences in non-glycosites. Normalization of gapped dipeptide value is performed using a min-max scaling. Finally, gapped dipeptides are assigned the corresponding value. Figure 10 depicts the sample 41-window motif, gapped dipeptide from N-terminal and C-terminal position, and corresponding normalized gapped dipeptide value. Feature vector length from the gapped dipeptide feature is 40 (=41 − 1 (central residue)). The features and their corresponding feature vector length for N-GlyDE, and N-GlycositeAtlas dataset are elaborated in Supplementary Table S4.

### 3.3. Overall Approach

**Figure 11:**
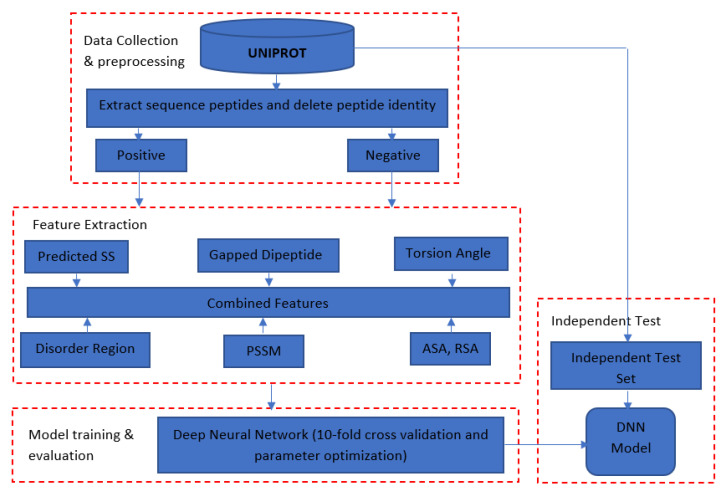
Schematic diagram showing the workflow used to develop the model. Secondary Structure (SS); Position Specific Scoring Matrix (PSSM); Accessible Surface Area (ASA); Relative Solvent Accessibility (RSA); Deep Neural Network (DNN).

DeepNGlyPred consists of four main steps (Figure 11). The first step is data collection and preprocessing in which protein sequences from the N-GlyDE and N-GlycositeAtlas datasets are downloaded from the UniProt database. The 25-residue window sequence for the N-GlyDE and 41-residue window sequence for the N-GlycositeAtlas dataset confined to the N-X-[S/T] sequons are extracted from the corresponding sites. Experimentally verified sites were considered as positive, whereas those sequons other than positive were considered as negative and were extracted from the same glycoproteins (for N-GlyDE). However, for the N-GlycositeAtlas dataset, positive annotated sites were taken from the N-GlycositeAtlas dataset and putative negative sites were obtained from the proteins that reside in the nucleus and mitochondrion. Furthermore, any overlapping motif within and across positive, negative training, and independent test set were removed. Moreover, window peptides that have more than 30% sequence identity were removed by CD-HIT. For the N-GlycositeAtlas dataset, since the number of negatives was higher than positives, the dataset was balanced using under-sampling.

In the second step, these window sequences (centered around the site of interest) are encoded with different encoding schemes as described in Section 3.2. In the third step (Model evaluation and training), individual feature importance analysis, as well as the group-wise combination of feature importance are performed. The best performing DNN architecture was obtained for N-GlyDE, and N-GlycositeAtlas dataset by grid search. Finally, in the fourth step, the trained model is evaluated using both 10-fold cross-validation on the training set and an independent test dataset and compared with different competitive predictors.

### 3.4. Model Training Using Deep Neural Network (DNN)

**Figure 12:**
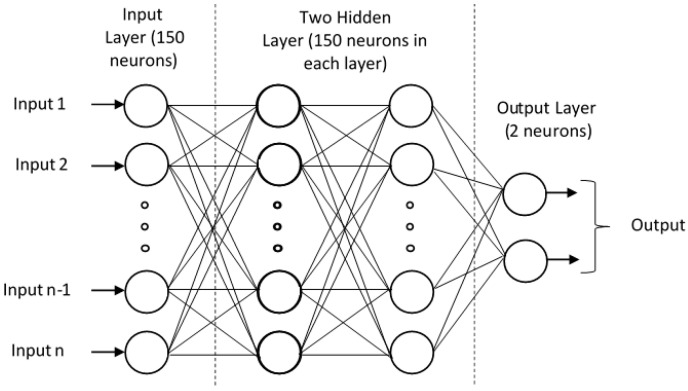
Schematic of Deep Neural network with 2 hidden layers for N-GlyDE dataset.

We performed our model training using Deep Neural Network (DNN, essentially a Multi-layer Perceptron (MLP)) as shown in Figure 12. The MLP is represented as a hierarchical (layered) organization of neurons (like the neurons in the brain) with connection to other neurons.

The DNN architecture is implemented in Python using Keras and Tensorflow modules. As elaborated in Table 1, and Table 2 the optimized parameters were obtained using a grid search. To avoid overfitting, we have used overfitting reduction techniques like dropout, early stopping, Model Checkpoint, and Reduce learning rate on the plateau. The loss and accuracy curve for training on N-GlyDE and N-GlycositeAtlas are shown in Supplementary Figures S3 and S4. It can be observed that no signs of underfitting and/or overfitting are present in the models.

### 3.5. Performance Evaluation

To evaluate the performance of each model, we use Accuracy, Sensitivity (SN), Specificity (SP), Matthews Correlation Coefficient (MCC), and Precision. The models were evaluated using 10-fold cross-validation on the benchmark training dataset and an independent test set.

ACC describes the correctly predicted residues out of the total residues (Equation (1)). Meanwhile, SN defines the model’s ability to distinguish positive residues (Equation (2)), and SP measures the model’s ability to correctly identify the negative residues (Equation (3)). Matthews Correlation Coefficient (MCC) is the calculated score that considers the model’s predictive capability concerning both positive and negative residues (Equation (4)). Likewise, precision reveals how many of the correctly predicted cases turned out to be positive (Equation (5)).

(1) $$Accuracy=\frac{TP+TN}{TP+TN+FP+FN}\times 100$$

(2) $$Sensitivity=\frac{TP}{TP+FN}\times 100$$

(3) $$Specificity=\frac{TN}{TN+FP}\times 100$$

(4) $$MCC=\frac{\left(TP\right)\left(TN\right)-\left(FP\right)\left(FN\right)}{\sqrt{\left(TP+FP\right)\left(TP+FN\right)\left(TN+FP\right)\left(TN+FN\right)}}$$

(5) $$Precision=\frac{TP}{TP+FP}\times 100$$

## 4. Conclusions

In this study, we developed DeepNGlyPred, a Deep Learning-based approach for accurate prediction of protein glycosylation sites confined to N-X-[S/T] for both positive and negative sequences. We developed two flavors of DeepNGlyPred based on the N-GlyDE dataset and N-GlycositeAtlas dataset. DeepNGlyPred uses sequence-based and structural-based features generated from NetSurfP-2.0, PSI-BLAST, and Gapped Dipeptide. Out of the three feature groups the feature from NetSurfP-2.0, i.e., accessible surface area, relative solvent accessibility, secondary structures, torsion angles (Φ, Ψ), disordered regions turned out to be the best discriminative features followed by position-specific scoring matrix and gapped dipeptide. Compared to other existing tools like N-GlyDE, GlycoMine, NetNGlyc, GlycoEP_Std_PPP, DeepNGlyPred performs better than these methods in terms of MCC. The better performance of DeepNGlyPred may be attributed to the larger training set, optimized Deep Learning architecture as well as the creation of the negative dataset (negative sites were selected from proteins residing in the nucleus and mitochondrion). In addition, we also created one of the largest non-redundant datasets to date for N-linked glycosylation prediction whose positive peptide sequons are extracted from N-linked glycosylated proteins while negative N-linked glycosylation peptides sequon are extracted from non-glycoproteins that reside at Nucleus and Mitochondrion from DeepLoc-1.0 database. N-linked glycosylation often also occurs as a co-translational process, in that regard one of the future works is to discriminate N-linked glycosylation sites from N-linked co-translational sites. Thus, DeepNGlyPred can be used to identify N-linked glycosylation sites efficiently and accurately.

Finally, all datasets and programs developed during this study have been made freely available to the bioinformatics community at https://github.com/dukkakc/DeepNGlyPred to further contribute towards the study of N-linked glycosylation.

## Figures and Tables

**Table 1:** Parameters used in DNN for N-GlyDE dataset.

Name of the Parameters	Parameters Used
No. of layers	4
No. of neuron in three layers	150
No. neuron in the output layer	2
Activation Function	sigmoid
Activation Function at output layer	softmax
Optimizer	Adam
Learning rate	0.001
Objective/loss function	Binary_crossentropy
Model Checkpoint	Monitor = ‘val_accuracy’
Reduce learning rate on plateau	Factor = 0.001
Early stopping	patience = 5
Dropout	0.3
Batch_size	256
Epochs	400

**Table 2:** Parameters used in DNN for N-GlycositeAtlas dataset.

Name of the Parameters	Parameters Used
No. of layers	5
No. neuron in four layers	1024
No. of neuron in the output layer	2
Activation Function	sigmoid
Activation Function at output layer	softmax
Optimizer	Adam
Learning rate	0.001
Objective/loss function	Binary_crossentropy
Model Checkpoint	Monitor = ‘val_accuracy’
Reduce learning rate on plateau	Factor = 0.001
Early stopping	patience = 5
Dropout	0.3
Batch_size	256
Epochs	400

**Table 3:** Prediction performance of DeepNGlyPred compared to other different predictors on the independent dataset.

Predictors	Accuracy (%)	Precision (%)	Sensitivity (%)	Specificity (%)	MCC
DeepNGlyPred (N-GlycositeAtlas)	79.4	66.9	88.6	73.9	0.605
DeepNGlyPred (N-GlyDE)	77.8	69.5	72.4	81.0	0.531
N-GlyDE	74.0	61.3	82.6	68.9	0.499
GlycoMine	72.5	61.6	70.0	73.9	0.43
NetNGlyc	57.2	46.0	84.4	41.1	0.265
GlycoEP_Std_PPP	57.4	43.7	51.2	61.0	0.119

**Table 4:** Positive and negative sites for training and testing.

Name of Dataset	Positive Site	Negative Site	Total
N-GlyDE (training)	1030	2050	3080
N-GlyDE (independent test)	167	280	447
N-GlycositeAtlas (training, CD-HIT)	9450	9450	18,900

## Data Availability

The datasets and codes for this study can be found at https://github.com/dukkakc/DeepNGlyPred.

## Supplementary Materials

The following are available online. Figure S1: Performance based on NetSurfP-2.0 features on different window sizes on the N-GlyDE dataset, Figure S2: Performance based on NetSurfP-2.0 features on different window sizes for N-GlycositeAtlas dataset, Figure S3: Loss and Accuracy curve when features extracted from NetSurfP-2.0, Gapped Dipeptide, PSI-BLAST (Position Specific Scoring Matrix) was fed into Deep Neural Network for N-GlyDE dataset, Figure S4: Loss and Accuracy curve when features extracted from NetSurfP-2.0, Gapped Dipeptide, PSI-BLAST (Position Specific Scoring Matrix) was fed into Deep Neural Network for N-GlycositeAtlas dataset, Figure S5: Ablation study to see the performance of DeepNGlyPred on various sizes of training data, Table S1: Ten-fold cross-validation on two training datasets for prediction of N-linked glycosylation sites by Deep Neural Network, Table S2: Performance measures when Xgboost feature extraction technique was used on N-GlyDE datasets to train the DNN and test on N-GlyDE independent test datasets, Table S3: Efficiency scores of individual and combined feature groups when trained on DNN with N-GlyDE training datasets and N-GlyDE independent test datasets, Table S4: Feature and Feature vector lengths, Table S5: Efficiency scores obtained from different Machine Learning models when trained with combination of NetSurfP-2.0, PSSM, and Gapped Dipeptide features at N-GlycositeAtlas data sets and tested on N-GlyDE independent data sets. We have optimized all the machine learning models. The Support Vector Machine was tested against two most important parameters, regularization constraint (C of 1 to 10), kernel (linear, rbf), SVM produced good results at C = 1 and kernel = ‘rbf’. Random Forest was tested against n_estimators: 50–300 in a delta of 50 and criterion: gini and entropy. Random Forest did best at n_estimators: 100 and criterion: entropy. Logistic Regression was tested against two important parameters: l1, l2, and solver: newton-cg, lbfgs, liblinear, sag, saga and it gave best result at penalty: l2 and solver: saga. For XGBoost after hyperparameter tuning we choose max_depth = 3, subsample = 0.8, n_estimators = 200, learning_rate = 0.05, random_state = 5. The naive bayes were tested on three variants, Multinomial, Bernoulli, Gaussian among them Gaussian was the good performer however it was slacking in performance compared to other machine learning models, Table S6: Efficiency scores obtained from different Deep Learning models when trained with combination of NetSurfP-2.0, PSSM, and Gapped Dipeptide features at N-GlycositeAtlas data sets and tested on N-GlyDE independent data sets, Table S7: Selection of window size for N-GlycositeAtlas dataset. The DNN was trained with 80% Training set, 10% validation set and tested on 10% independent training set, Table S8: Efficiency scores obtained from SPRINT-Gly when independent dataset is fed into the server.

## References

[1] Ohtsubo K., Marth J.D. (2006). Glycosylation in Cellular Mechanisms of Health and Disease. Cell.

[2] Aebi M., Bernasconi R., Clerc S., Molinari M. (2010). N-glycan structures: Recognition and processing in the ER. Trends Biochem. Sci..

[3] Lederkremer G.Z. (2009). Glycoprotein folding, quality control and ER-associated degradation. Curr. Opin. Struct. Biol..

[4] Varki A., Lowe J.B., Varki A., Cummings R.D. (2009). Biological Roles of Glycans. Essentials of Glycobiology.

[5] Schwarz F., Aebi M. (2011). Mechanisms and principles of N-linked protein glycosylation. Curr. Opin. Struct. Biol..

[6] Gavel Y., von Heijne G., Creaser E., Murali C., Britt K. (1990). Sequence differences between glycosylated and non-glycosylated Asn-X-Thr/Ser acceptor sites: Implications for protein engineering. Protein Eng..

[7] Boscher C., Dennis J.W., Nabi I.R. (2011). Glycosylation, galectins and cellular signaling. Curr. Opin. Cell Biol..

[8] van Kooyk Y., Rabinovich G.A. (2008). Protein-glycan interactions in the control of innate and adaptive immune responses. Nat. Immunol..

[9] Pérez-Sala D., Mollinedo F. (1995). Inhibition of N-linked glycosylation induces early apoptosis in human promyelocytic HL-60 cells. J. Cell. Physiol..

[10] Woods R.J., Edge C.J., Dwek R.A. (1994). Protein surface oligosaccharides and protein function. Nat. Genet. Mol. Biol..

[11] Wormald M.R., Dwek R.A. (1999). Glycoproteins: Glycan presentation and protein-fold stability. Structure.

[12] Ou X., Liu Y., Lei X., Li P., Mi D., Ren L., Guo L., Guo R., Chen T., Hu J. (2020). Characterization of spike glycoprotein of SARS-CoV-2 on virus entry and its immune cross-reactivity with SARS-CoV. Nat. Commun..

[13] Hennet T. (2012). Diseases of glycosylation beyond classical congenital disorders of glycosylation. Biochim. Biophys. Acta.

[14] Jaeken J., Rymen D., Matthijs G. (2013). Congenital disorders of glycosylation: Other causes of ichthyosis. Eur. J. Hum. Genet..

[15] Zhang H., Chan D.W. (2007). Cancer Biomarker Discovery in Plasma Using a Tissue-targeted Proteomic Approach. Cancer Epidemiol. Biomark. Prev..

[16] Kowarik M., Young N.M., Numao S., Schulz B.L., Hug I., Callewaert N., Mills D.C., Watson D.C., Hernandez M., Kelly J.F. (2006). Definition of the bacterial N-glycosylation site consensus sequence. EMBO J..

[17] Petrescu A.-J., Milac A.-L., Petrescu S.M., Dwek R.A., Wormald M.R. (2003). Statistical analysis of the protein environment of N-glycosylation sites: Implications for occupancy, structure, and folding. Glycobiology.

[18] Zielinska D.F., Gnad F., Wiśniewski J.R., Mann M. (2010). Precision Mapping of an In Vivo N-Glycoproteome Reveals Rigid Topological and Sequence Constraints. Cell.

[19] Schulz B.L., Petrescu S. (2012). Beyond the Sequon: Sites of N-Glycosylation. Glycosylation.

[20] Nita-Lazar M., Wacker M., Schegg B., Amber S., Aebi M. (2004). The N-X-S/T consensus sequence is required but not sufficient for bacterial N-linked protein glycosylation. Glycobiology.

[21] Wacker M., Feldman M., Callewaert N., Kowarik M., Clarke B.R., Pohl N.L., Hernandez M., Vines E.D., Valvano M., Whitfield C. (2006). Substrate specificity of bacterial oligosaccharyltransferase suggests a common transfer mechanism for the bacterial and eukaryotic systems. Proc. Natl. Acad. Sci. USA.

[22] Medzihradszky K.F. (2005). Peptide Sequence Analysis. Methods Enzymol..

[23] Agarwal K.L., Kenner G.W., Sheppard R.C. (1969). Feline gastrin. An example of peptide sequence analysis by mass spectrometry. J. Am. Chem. Soc..

[24] Slade D., Subramanian V., Fuhrmann J., Thompson P.R. (2013). Chemical and biological methods to detect post-translational modifications of arginine. Biopolymers.

[25] Gupta R., Brunak S. (2001). Prediction of glycosylation across the human proteome and the correlation to protein function. Pac. Symp. Biocomput..

[26] Caragea C., Sinapov J., Silvescu A., Dobbs D., Honavar V. (2007). Glycosylation site prediction using ensembles of Support Vector Machine classifiers. BMC Bioinform..

[27] Chauhan J.S., Bhat A.H., Raghava G.P.S., Rao A. (2012). GlycoPP: A Webserver for Prediction of N- and O-Glycosites in Prokaryotic Protein Sequences. PLoS ONE.

[28] Chien C.-H., Chang C.-C., Lin S.-H., Chen C.-W., Chang Z.-H., Chu Y.-W. (2020). N-GlycoGo: Predicting Protein N-Glycosylation Sites on Imbalanced Data Sets by Using Heterogeneous and Comprehensive Strategy. IEEE Access.

[29] Pugalenthi G., Nithya V., Chou K.-C., Archunan G. (2020). Nglyc: A Random Forest Method for Prediction of N-Glycosylation Sites in Eukaryotic Protein Sequence. Protein Pept. Lett..

[30] Li F., Li C., Wang M., Webb G., Zhang Y., Whisstock J.C., Song J. (2015). GlycoMine: A machine learning-based approach for predicting N-, C- and O-linked glycosylation in the human proteome. Bioinformatics.

[31] Taherzadeh G., Dehzangi A., Golchin M., Zhou Y., Campbell M.P. (2019). SPRINT-Gly: Predicting N- and O-linked glycosylation sites of human and mouse proteins by using sequence and predicted structural properties. Bioinformatics.

[32] Adamczak R., Porollo A., Meller J. (2004). Accurate prediction of solvent accessibility using neural networks-based regression. Proteins.

[33] McGuffin L.J., Bryson K., Jones D.T. (2000). The PSIPRED protein structure prediction server. Bioinformatics.

[34] Petersen B., Petersen T.N., Andersen P., Nielsen M., Lundegaard C. (2009). A generic method for assignment of reliability scores applied to solvent accessibility predictions. BMC Struct. Biol..

[35] Heffernan R., Yang Y., Paliwal K.K., Zhou Y. (2017). Capturing non-local interactions by long short-term memory bidirectional recurrent neural networks for improving prediction of protein secondary structure, backbone angles, contact numbers and solvent accessibility. Bioinformatics.

[36] Ward J.J., McGuffin L., Bryson K., Buxton B.F., Jones D.T. (2004). The DISOPRED server for the prediction of protein disorder. Bioinformatics.

[37] Ward J., Sodhi J., McGuffin L., Buxton B., Jones D. (2004). Prediction and Functional Analysis of Native Disorder in Proteins from the Three Kingdoms of Life. J. Mol. Biol..

[38] Hanson J., Yang Y., Paliwal K.K., Zhou Y. (2016). Improving protein disorder prediction by deep bidirectional long short-term memory recurrent neural networks. Bioinformatics.

[39] Li F., Li C., Revote J., Zhang Y., Webb G.I., Li J., Song J., Lithgow T. (2016). GlycoMinestruct: A new bioinformatics tool for highly accurate mapping of the human N-linked and O-linked glycoproteomes by incorporating structural features. Sci. Rep..

[40] Pitti T., Chen C.-T., Lin H.-N., Choong W.-K., Hsu W.-L., Sung T.-Y. (2019). N-GlyDE: A two-stage N-linked glycosylation site prediction incorporating gapped dipeptides and pattern-based encoding. Sci. Rep..

[41] Sun S., Hu Y., Ao M., Shah P., Chen J., Yang W., Jia X., Tian Y., Thomas S., Zhang H. (2019). N-GlycositeAtlas: A database resource for mass spectrometry-based human N-linked glycoprotein and glycosylation site mapping. Clin. Proteom..

[42] Do D.T., Le T.Q., Le N.Q. (2020). Using deep neural networks and biological subwords to detect protein S-sulfenylation sites. Brief. Bioinform..

[43] Thapa N., Chaudhari M., McManus S., Roy K., Newman R.H., Saigo H., Kc D.B. (2020). DeepSuccinylSite: A deep learning based approach for protein succinylation site prediction. BMC Bioinform..

[44] Thapa N., Chaudhari M., Iannetta A.A., White C., Roy K., Newman R.H., Hicks L.M., Kc D.B. (2021). A deep learning based approach for prediction of Chlamydomonas reinhardtii phosphorylation sites. Sci. Rep..

[45] Pakhrin S., Shrestha B., Adhikari B., Kc D. (2021). Deep Learning-Based Advances in Protein Structure Prediction. Int. J. Mol. Sci..

[46] Klausen M.S., Jespersen M.C., Nielsen H., Jensen K.K., Jurtz V.I., Sønderby C.K., Sommer M.O.A., Winther O., Nielsen M., Petersen B. (2019). NetSurfP-2.0: Improved prediction of protein structural features by integrated deep learning. Proteins.

[47] Altschul S.F., Madden T.L., Schäffer A.A., Zhang J., Zhang Z., Miller W., Lipman D.J. (1997). Gapped BLAST and PSI-BLAST: A new generation of protein database search programs. Nucleic Acids Res..

[48] Armenteros J.J.A., Sønderby C.K., Sønderby S.K., Nielsen H., Winther O. (2017). DeepLoc: Prediction of protein subcellular localization using deep learning. Bioinformatics.

[49] Lemaitre G., Nogueira F., Aridas C.K. (2017). Imbalanced-learn: A Python Toolbox to Tackle the Curse of Imbalanced Datasets in Machine Learning. J. Mach. Learn. Res..

[50] Li W., Godzik A. (2006). Cd-hit: A fast program for clustering and comparing large sets of protein or nucleotide sequences. Bioinformatics.

[51] Crooks G.E., Hon G., Chandonia J.-M., Brenner S.E. (2004). WebLogo: A Sequence Logo Generator. Genome Res..

[52] van der Maaten L., Hinton G. (2008). Visualizing Data using t-SNE. J. Mach. Learn. Res..

