# Supplementary material for: DeepNGlyPred: A Deep Neural Network-Based Approach for Human N-Linked Glycosylation Site Prediction
Source: Molecules. 2021 Dec 2;26(23):7314. doi: 10.3390/molecules26237314 (PMC8658957; doi:10.3390/molecules26237314)
Supplement: Supplementary file 1 [file molecules-26-07314-s001.zip › molecules-1466942-supplementary.pdf]

Supplementary Section:

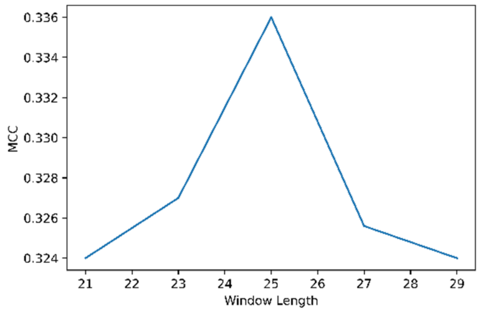

Figure S1: Performance based on NetSurfP-2.0 features on different window sizes on the N-GlyDE dataset.

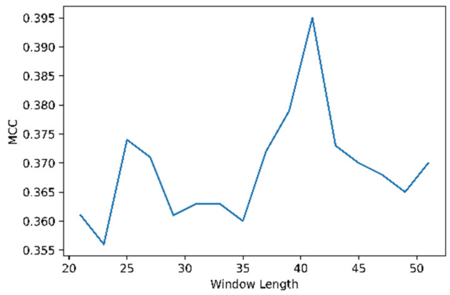

Figure S2: Performance based on NetSurfP-2.0 features on different window sizes for N-GlycositeAtlas dataset.

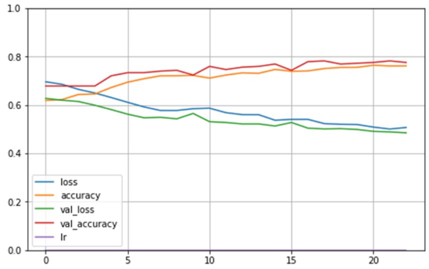

Figure S3: Loss and Accuracy curve when features extracted from NetSurfP-2.0, Gapped Dipeptide, PSI-BLAST (Position Specific Scoring Matrix) was fed into Deep Neural Network for N-GlyDE dataset.

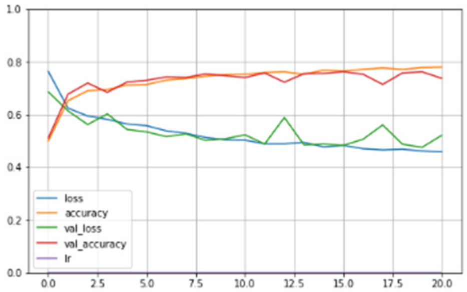

Figure S4: Loss and Accuracy curve when features extracted from NetSurfP-2.0, Gapped Dipeptide, PSI-BLAST (Position Specific Scoring Matrix) was fed into Deep Neural Network for N-GlycositeAtlas dataset.

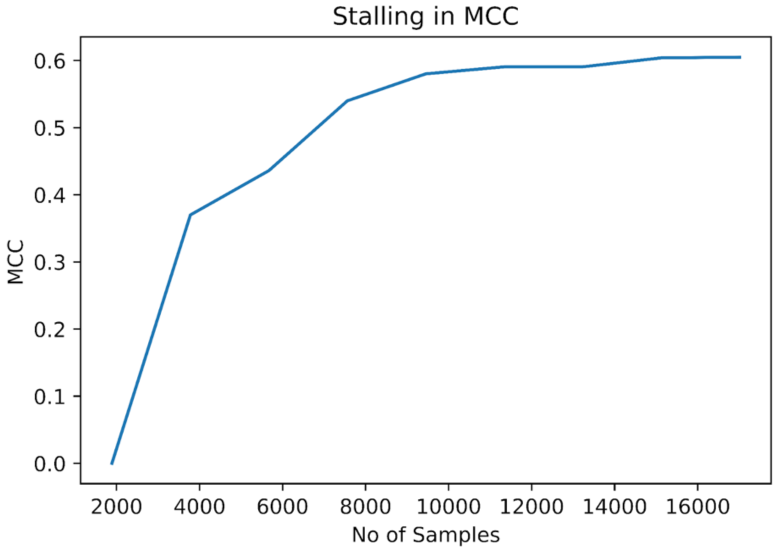

Figure S5: Ablation study to see the performance of DeepNGlyPred on various sizes of training data.

Table S1: Ten-fold cross-validation on two training datasets for prediction of N-linked glycosylation sites by Deep Neural Network.

Dataset	MCC ± SD	Specificity ± SD	Sensitivity ± SD	Accuracy ± SD

N-GlycositeAtlas (Window 41)	0.5197 ± 0.0305	0.8231 ± 0.00963	0.6819 ± 0.0858	0.7532 ± 0.0127
N-GlyDE (Window 25)	0.4440 ± 0.0499	0.4449 ± 0.1020	0.9272 ± 0.0338	0.7662 ± 0.0202

Table S2: Performance measures when Xgboost feature extraction technique was used on N-GlyDE datasets to train the DNN and test on N-GlyDE independent test datasets.

Dataset	MCC	Accuracy	Specificity	Sensitivity / Recall	Dimension Reduction
N-GlyDE	0.5059	0.76	0.77	0.73	3,028 to 1,118

Table S3: Efficiency scores of individual and combined feature groups when trained on DNN with N-GlyDE training datasets and N-GlyDE independent test datasets.

Feature	MCC	Accuracy	Specificity	Sensitivity	Precision
NetSurfP-2.0	0.414	0.72	0.76	0.65	0.62
PSSM	0.327	0.66	0.68	0.66	0.54
Gapped Dipeptide	0.315	0.68	1	0.149	1.0
NetSurfP-2.0 + PSSM	0.45	0.718	0.67	0.79	0.59
NetSurfP-2.0 + PSSM + Gapped Dipeptide	0.57	0.80	0.8571	0.7065	0.7468

Table S4: Feature and Feature vector lengths.

Feature Name	Feature Vector Length (N-glycositeAtlas)	Feature Vector Length (N-GlyDE)
NetSurfP - 2.0	328	200
PSSM	820	500
Gapped Dipeptide	40	24

Table S5: Efficiency scores obtained from different Machine Learning models when trained with combination of NetSurfP – 2.0, PSSM, and Gapped Dipeptide features at N-GlycositeAtlas data sets and tested on N-GlyDE independent data sets. We have optimized all the machine learning models. The Support Vector Machine was tested against two most important parameters, regularization constraint (C of 1 to 10), kernel (linear, rbf), SVM produced good results at C=1 and kernel = 'rbf'. Random Forest was tested against n_estimators: 50-300 in a delta of 50 and criterion: gini and entropy. Random Forest did best at n_estimators: 100 and criterion: entropy. Logistic Regression was tested against two important parameters: l1, l2, and solver: newton-cg, lbfgs, liblinear, sag, saga and it gave best result at penalty: l2 and solver: saga. For XGBoost after hyperparameter tuning we choose max_depth = 3, subsample = 0.8, n_estimators = 200, learning_rate = 0.05, random_state = 5. The naive bayes were tested on three variants, Multinomial, Bernoulli, Gaussian among them Gaussian was the good performer however it was slacking in performance compared to other machine learning models.

Machine Learning Model	MCC	Accuracy	Specificity	Sensitivity	Precision

Logistic Regression	0.5279	0.74	0.657	0.886	0.606
Support Vector Machine	0.5178	0.76	0.850	0.653	0.778
XGBoost	0.4456	0.69	0.592	0.862	0.558
Random Forest	0.4361	0.68	0.560	0.880	0.544
Gaussian Naive Bayes	0.1226	0.44	0.171	0.916	0.397

Table S6: Efficiency scores obtained from different Deep Learning models when trained with combination of NetSurfP – 2.0, PSSM, and Gapped Dipeptide features at N-GlycositeAtlas data sets and tested on N-GlyDE independent data sets.

Deep Learning Model	MCC	Accuracy	Specificity	Sensitivity	Precision
ResNet	0.564	0.77	0.721	0.862	0.862
Convolution 1D	0.570	0.76	0.664	0.922	0.922
Convolution 2D	0.546	0.77	0.775	0.754	0.784
UNet	0.519	0.74	0.660	0.874	0.874
LSTM	0.566	0.79	0.832	0.736	0.736
BiLSTM	0.574	0.79	0.794	0.796	0.790

Table S7: Selection of window size for N-GlycositeAtlas dataset. The DNN was trained with 80% Training set, 10 % validation set and tested on 10 % independent training set.

Window Size	MCC	Accuracy	Precision	Sensitivity	Specificity
21	0.361	0.664	0.772	0.493	0.846
23	0.356	0.678	0.678	0.712	0.643
25	0.374	0.685	0.716	0.642	0.73
27	0.371	0.685	0.672	0.757	0.609
29	0.361	0.673	0.736	0.568	0.785
31	0.363	0.678	0.723	0.604	0.755
33	0.363	0.676	0.731	0.586	0.772
35	0.36	0.673	0.735	0.569	0.782
37	0.372	0.686	0.701	0.678	0.694
39	0.379	0.684	0.739	0.595	0.778
41	0.395	0.695	0.728	0.650	0.743
43	0.373	0.675	0.762	0.534	0.824
45	0.37	0.68	0.733	0.593	0.772
47	0.368	0.682	0.662	0.78	0.58
49	0.365	0.678	0.728	0.595	0.765
51	0.37	0.679	0.739	0.579	0.784

Table S8: Efficiency scores obtained from SPRINT-Gly when independent dataset is fed into the server.

Predictor	MCC	Accuracy	Specificity	Sensitivity	Precision
SPRINT-Gly	0.03656	0.3758	0.0035	1	0.374

Table S9. McNemar's Test, Comparison between DeepNGlyPred and different Machine Learning Classifiers

ML classifier	P value	Accept H0	Reject H0
Logistic Regression	0.716	√	
XGBoost	0.001		√
Gaussian Naïve Bayes	0.000		√
Random Forest	0.000		√
SVM	0.070	√	

Table S10. McNemar's Test, Comparison between DeepNGlyPred and different Deep Learning architecture

DL Classifier	P value	Accept H0	Reject H0
LSTM	0.000		√
BiLSTM	0.001		√
2D Convolution	0.000		√
1D Convolution	0.045		√
ResNet	1.000	√	
UNet	0.045		√
